# Conduction Velocity of Muscle Action Potential of Knee Extensor Muscle During Evoked and Voluntary Contractions After Exhaustive Leg Pedaling Exercise

**DOI:** 10.3389/fphys.2020.00546

**Published:** 2020-05-27

**Authors:** Kohei Watanabe, Taiki Sakai, Shosaku Kato, Natsuka Hashizume, Naoki Horii, Maki Yoshikawa, Natsuki Hasegawa, Keiko Iemitsu, Katsunori Tsuji, Masakata Uchida, Masao Kanamori, Motoyuki Iemitsu

**Affiliations:** ^1^Laboratory of Neuromuscular Biomechanics, School of International Liberal Studies, Chukyo University, Nagoya, Japan; ^2^Faculty of Sport and Health Science, Ritsumeikan University, Kusatsu, Japan; ^3^Research Fellow of Japan Society for the Promotion of Science, Tokyo, Japan; ^4^Research Organization of Science and Technology, Ritsumeikan University, Kusatsu, Japan

**Keywords:** neuromuscular fatigue, quadriceps, cycling, vastus lateralis, multi-channel surface electromyography

## Abstract

**Purpose:**

Muscle fiber conduction velocity (CV) has been developed to estimate neuromuscular fatigue and measured during voluntary (VC) and electrically evoked (EC) contractions. Since CV during VC and EC reflect different physiological phenomena, the two parameters would show inconsistent changes under the conditions of neuromuscular fatigue. We investigated the time-course changes of CV during EC and VC after fatiguing exercise.

**Methods:**

In 14 young males, maximal voluntary contraction (MVC) of knee extensor muscles, CV during electrical stimulation (CV-EC) and MVC (CV-VC) were measured before and immediately, 30 min, 60 min, 120 min, and 24 h after exhaustive leg pedaling exercise.

**Results:**

CV-EC significantly increased immediately after the fatiguing exercise (*p* < 0.05) and had a significant negative correlation with MVC in merged data from all time-periods (*r* = −0.511, *p* < 0.001). CV-VC significantly decreased 30, 60, and 120 min after the fatiguing exercise (*p* < 0.05) and did not show any correlations with MVC (*p* > 0.05).

**Conclusion:**

These results suggest that CV during EC and VC exhibits different time-course changes, and that CV during EC may be appropriate to estimate the degree of neuromuscular fatigue after fatiguing pedaling exercise.

## Introduction

Muscle fiber conduction velocity (CV) recorded by multi-channel surface electromyography (EMG) has been used to assess muscular fatigue during and/or after exercise (Merletti et al., 1990). Decreases in CV during and/or after fatiguing exercise were noted in previous studies, and this phenomenon has been explained by fatigue-induced physiological responses of peripheral muscle, such as the accumulation of metabolites or a decrease in the membrane potential (Mortimer et al., 1970; Moxham et al., 1982; Milner-Brown and Miller, 1986). Regarding assessment of neuromuscular fatigue in actual sporting events, CV during voluntary contraction (VC) has been measured (Boccia et al., 2017a, b). Since this method can simultaneously assess fatigue-induced declines in the force generation capacity, such as maximal voluntary contraction (MVC) force, and does not require any additional measurement system except for recording EMG and exerted force, it may be useful for field measurements. Boccia et al. (2017a) applied this technique to assess neuromuscular fatigue and demonstrated a decrease in CV after a half-marathon run and positive correlation between exercise-induced decreases in MVC and CV (Boccia et al., 2017a). On the other hand, Schmitz et al. (2012) reported that no decrease in CV was detected during exhaustive bicycling (Schmitz et al., 2012). This difference in results may be partly explained by not only task-dependent muscular fatigue but also the use of surface EMG during VC to calculate CV. Surface EMG signals measured during VC include information from both central and peripheral factors, since muscle action potentials during VC originate from rate coding and the recruitment of motor units regulated by the central nervous system (CNS). Therefore, it would be difficult to separately assess peripheral muscular fatigue and physiological responses in the CNS based on CV calculated from surface EMG signals during VC (Merletti et al., 1990; Minetto et al., 2010). For the separation of muscular factors, muscle contractions evoked by neuromuscular electrical stimulations have been used for the calculation of CV (Minetto et al., 2010; Piitulainen et al., 2011). Under constant neuromuscular stimulation, changes in CV can be explained by alterations in peripheral muscular factors. Piitulainen et al. (2011) showed a decrease in CV during electrically evoked contraction (EC) 30 and 120 min after repeated eccentric contractions during elbow flexion (Piitulainen et al., 2011). They also reported that decreases in CV were not detected after repeated concentric contractions during elbow flexion, meaning that CV could reflect task-dependent muscle fatigue. Moreover, this study measured CV during MVC and the increase in CV immediately after repeated concentric contractions. This means that different patterns are observed in CV calculated from surface EMG during VC and EC. While CV detection during EC could be useful to assess peripheral muscular fatigue, applications of this method to the multiple joint dynamic exercises generally used in physical training such as running and/or pedaling have yet to be investigated.

The aim of this study was to investigate the time-course changes in CV of knee extensor muscle during EC and VC after fatiguing pedaling exercise. A previous study by Piitulainen et al. (2011) demonstrated an unchanged CV during EC and increased CV during VC after repeated concentric contractions. We hypothesized that CV during EC remains unchanged but that during VC increases after pedaling exercise, which mainly involves concentric contraction of knee and hip extensor muscles. While CV has been used to assess neuromuscular fatigue, this parameter is sensitive to changes in muscle temperature (Petrofsky and Lind, 1980; Petrofsky and Laymon, 2005). Although it is difficult to control muscle temperature during and after exercise, simultaneous recording of direct or indirect muscle temperature would be necessary to correctly interpret the results of CV. We thus also hypothesized that individual differences in CV changes could be explained by an indicator of muscle temperature when the indicator is altered by exercise.

## Materials and Methods

### Participants

Fourteen healthy young men (age: 22.3 ± 0.3 years, height: 171.8 ± 7.3 cm, body mass: 61.1 ± 6.8 kg, VO2 peak: 48 ± 5 ml/kg/min) volunteered for the present study. All subjects gave written informed consent for the study after receiving a detailed explanation of the purposes, potential benefits, and risks associated with participation. All subjects were healthy with no history of any musculoskeletal or neurological disorders. All study procedures were conducted in accordance with the Declaration of Helsinki and research code of ethics of Chukyo University, and were approved by the Committee for Human Experimentation of Ritsumeikan University (No. BKC-2017-074).

### Experimental Design

**FIGURE 1:**
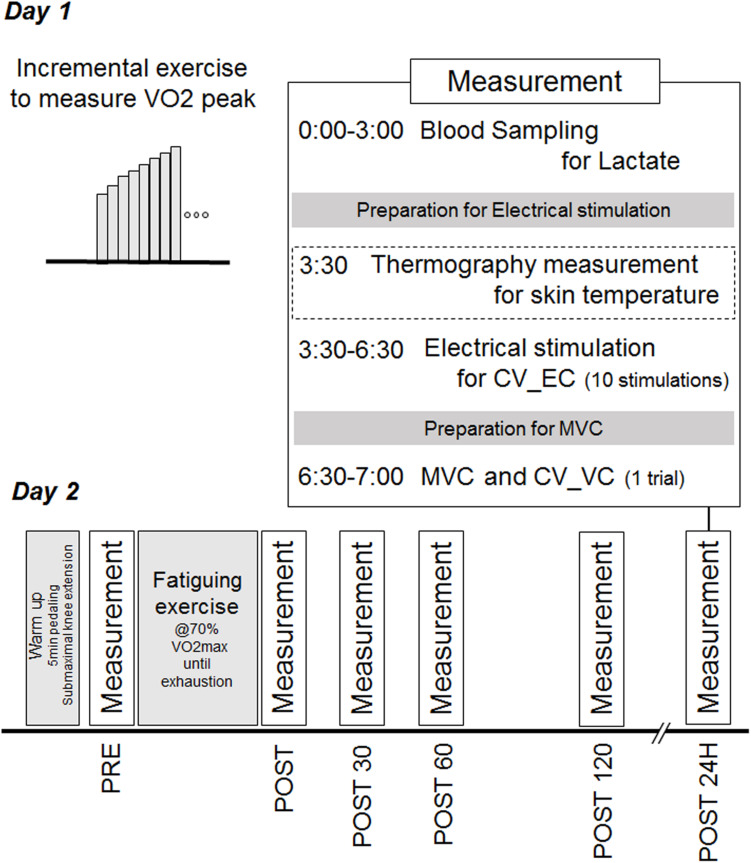
Time schedule of the exercises and measurements in this study.

Participants performed incremental exercise test to identify peak oxygen consumption (VO2 max) and fatiguing pedaling exercise on a cycle ergometer on the separated days. To assess neuromuscular fatigue, MVC and surface EMG signals during EC and MVC for the calculation of CV before (PRE) were recorded for knee extensor muscles, immediately after (POST), 30 min after (POST30), 60 min after (POST60), 120 min after (POST120), and 24 h after (POST24h) the fatiguing exercise. Rest intervals between EC and MVC was approximately 1 min. At each time-point, the blood lactate concentration was also measured with the lactate oxidase method using an automated analyzer (Lactate Pro 2, Arkray, Kyoto, Japan). The temperature of skin directly overlying the vastus lateralis muscle was measured in 10 of 14 participants using a thermography (TVS-8502, Nippon Avionics Co., Ltd., Tokyo, Japan). Time schedule of this study was shown in Figure 1.

### Incremental Exercise Test

At least 7 days before the trial, each participant performed an exercise tolerance test to determine peak oxygen consumption (VO2 peak). Participants performed incremental pedaling exercise on a cycle ergometer (Monark 828E, Monark Exercise AB, Vansbro, Sweden) at a cadence of 60 bpm. The workload during this tolerance test was increased by 15 W every 1 min starting from 75 to 90 W. We set three criteria to determine VO2 max: (1) heart rate reached the prospective maximal value [220 – age (bpm)], (2) oxygen uptake (VO2) reached a steady-state despite increasing workloads, or (3) participants could not maintain the pedaling at 60 bpm.

### Fatiguing Exercise

As fatiguing exercise, the participants performed constant pedaling on the cycle ergometer at 70% of VO2 max until exhaustion, i.e., participants could not maintain the pedaling at 60 bpm.

### Maximal Voluntary Contraction

The participants were seated comfortably with the right leg fixed in a custom-made dynamometer (Takei Scientific Instruments Co., Ltd., Niigata, Japan) with a force transducer (LU-100KSE; Kyowa Electronic Instruments, Tokyo, Japan) and both hip and knee joint angles flexed at 90° (180°corresponds to full extension) using our previously reported procedure (Watanabe et al., 2016). The participants were asked to gradually increase their knee extension force from the baseline to maximum in 2–3 s and then sustain it maximally for 2 s. As a warm-up and for familiarization, the participants performed 5 min of pedaling exercise at a very low workload and with submaximal contractions at approximately 50, 70, and 90% of maximal contractions and one MVC 5 min before PRE measurements.

### Surface EMG and CV Calculation

**FIGURE 2:**
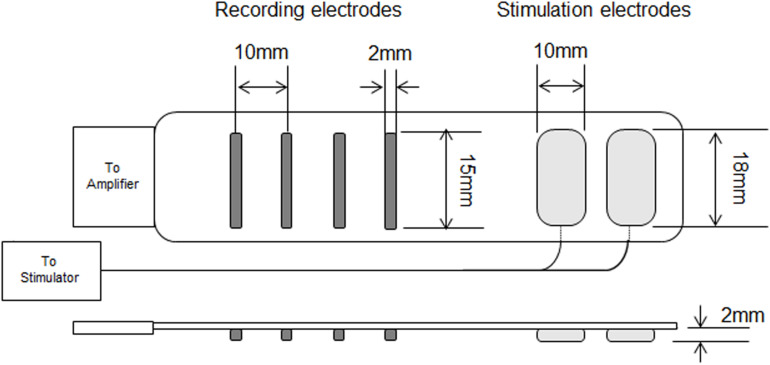
Recording and stimulation electrodes used in this study.

Surface EMG signals were recorded from the vastus lateralis muscle with a specially designed electrode system including a four linear electrode array for recording EMG and two electrodes for electrical stimulation (SMK Corporation, Tokyo, Japan) (Patent applications: 2016-113372 in Japan and 2017/0347908 A1 in the United States). Each recording electrode has a 2 × 15-mm detection area and their inter-electrode distance is 10 mm. Stimulation electrodes (10 × 18 mm) are located along the same line as the recording electrode array and their inter-electrode distance is 15 mm. The distance between the nearest stimulation and recording electrodes is 22 mm (Figure 2). Recording and stimulation electrodes were connected to a surface EMG amplifier (EMG-USB 2+, OT Bioelettronica, Torino, Italy) and an electrical stimulator (MEB-9400, Nihon Kohden, Tokyo, Japan), respectively. The detection area of electrode for measuring surface EMG were made with gold-plated. Surface EMG was sampled at 2,048 Hz with a band-pass filter (10–450 Hz). Before fixation of the electrode system by double-coated adhesive tape, the skin surface was shaved and cleaned with alcohol, and a process to determine the electrode location and arrangement was performed. Lateral and distal sites of the thigh and the motor point of the vastus lateralis muscle were searched for using electrical stimulation at 1 Hz. The electrode arrangement was determined based on clearly propagated muscle fiber action potentials during electrical stimulations. The determined electrode position was marked on the skin surface. After the measurements at PRE and POST120, the electrode system was removed and replaced before the measurements at POST and POST24h, relying on the skin-surface markings.

For the calculation of CV during EC (CV-EC), 10 continuous electrical stimulations with a 0.5-ms duration and 1-Hz stimulation frequency were applied at the estimated motor point before MVC at each time-point with 50 mA of stimulation intensity. CV of surface EMG was calculated from two pairs of two adjacent differential surface EMG signals with a 1-s time window using previously described algorithms (Merletti et al., 1990). We confirmed the accuracy of CV calculation based on the correlation coefficient between the signals and visual inspection of signal propagation. For CV during EC, one of ten elicited action potentials with highest correlation coefficient value for calculation of CV was chosen and used for further analysis. CV during VC (CV-VC) was calculated with a 1-s time window when the peak force was observed during MVC. The same process was applied for the calculation of CV-EC and CV-VC.

Since it is well-known that CV is influenced by muscle temperature, the skin temperature near the recording electrodes was recorded using a thermography (TVS-8502, Nippon Avionics Co., Ltd., Tokyo, Japan) to estimate changes in muscle temperature during the experiments. The distance between thermography camera and the participants was 2 m. Skin temperatures from five points around electrode location on the vastus lateralis muscle were measured just before the measurements of CV-EC and averaged values from the five measured points were used for further analysis. Room temperature was set at under the neutral temperature environment (Crawford et al., 2006), but not strictly controlled and average of room temperature was 24.1 ± 1.3°C.

### Statistical Analysis

All data are presented as the mean and standard deviation (SD). The present study used non-parametric tests because of the small sample size. We used the Friedman test to investigate the effect of the time-course on CV-EC, CV-VC, MVC, blood lactate concentration, and skin temperature. When a significant time-course effect was detected, Dunn’s test with Bonferroni correction was performed. Associations of CV-EC and CV-VC with the skin temperature and MVC (relative values) were calculated for each time-point and compared with the values at PRE and merged data using Spearman’s rank correlation coefficient. The level of significance was set at *p* < 0.05. Statistical analysis was performed using IBM SPSS statistics 21 (IBM, Chicago, United States).

## Results

**FIGURE 3:**
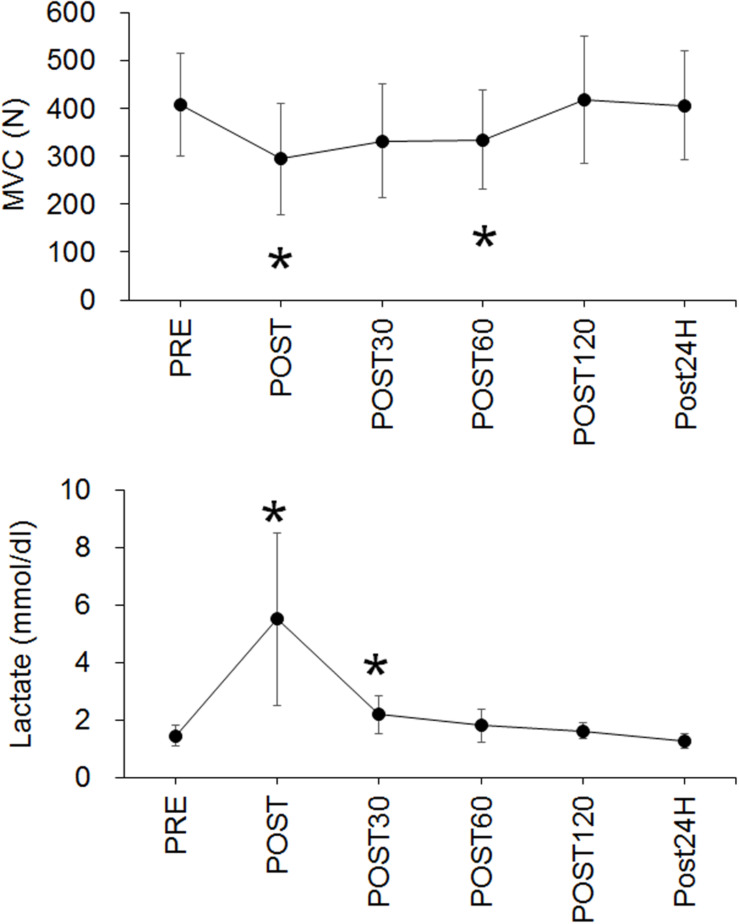
Time-course changes in maximal voluntary contraction (MVC) (upper panel) and blood lactate concentration (Lactate) (lower panel). *p < 0.05 vs. PRE.

The workload at VO2 peak and for exhaustive exercise were 235 ± 34 W and 160 ± 22 W in the present study, respectively. The time to exhaustion with the fatiguing exercise was 36.4 ± 10.0 min. A significant effect of time on MVC was noted (*p* < 0.001) and the values at POST (*p* = 0.005) and POST60 (*p* = 0.045) were significantly lower than that at PRE (Figure 3). The blood lactate concentration also significantly changed over time (*p* < 0.001), with the values being significantly higher at POST (*p* = 0.005) and POST30 (*p* = 0.01) when compared with that at PRE (Figure 3).

**FIGURE 4:**
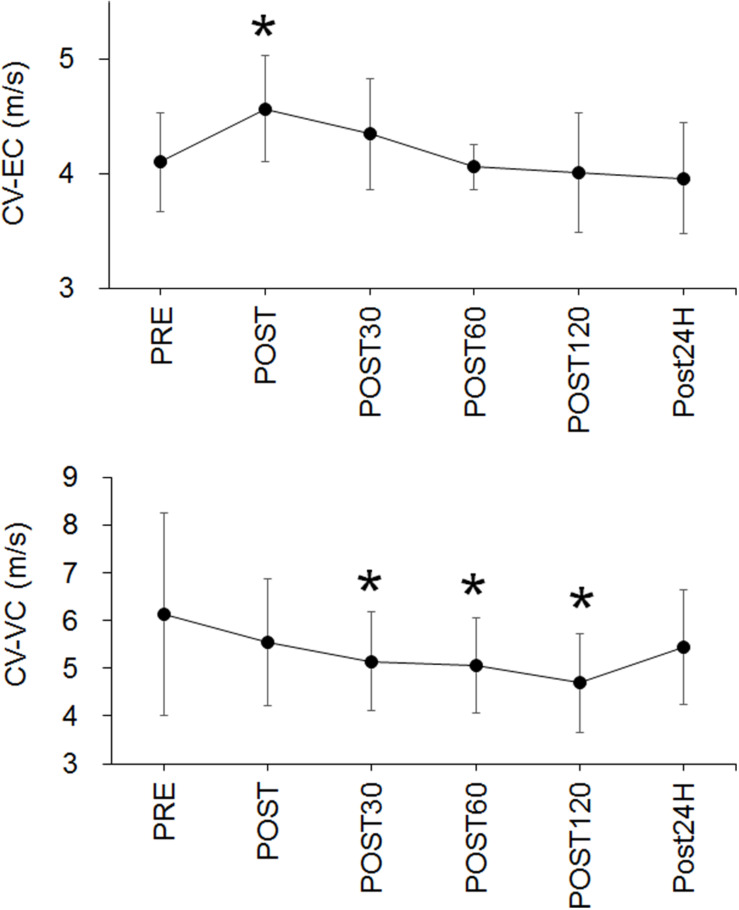
Time-course changes in conduction velocity during evoked contraction (CV-EC) (upper panel) and conduction velocity during voluntary contraction (CV-VC) (lower panel). *p < 0.05 vs. PRE.

For CV-EC, a significant time effect (*p* < 0.001) and significant increase at POST (*p* = 0.005) were noted (Figure 4). For CV-VC, a significant time effect (*p* < 0.001) and significant decreases at POST30 (*p* = 0.03), POST60 (*p* = 0.005), and POST120 (*p* = 0.005) were identified (Figure 4).

**FIGURE 5:**
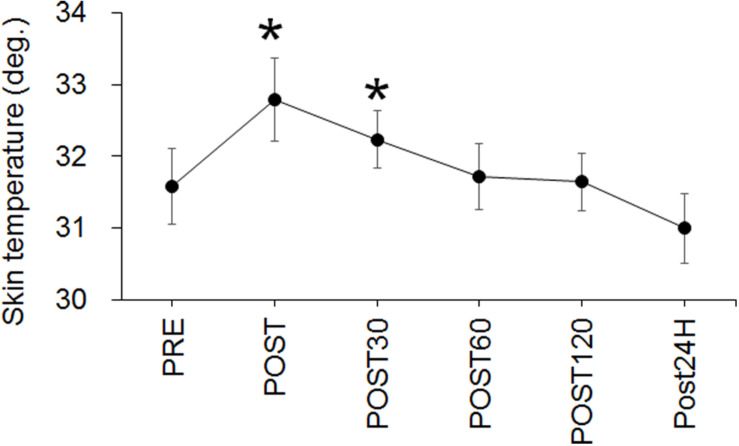
Time-course changes in skin temperature. *p < 0.05 vs. PRE.

The skin temperature significantly changed over time (*p* < 0.001) and the values at POST (*p* = 0.005) and POST30 (*p* = 0.03) were significantly higher than that at PRE (Figure 5). A negative correlation between CV-EC and skin temperature was seen at POST120 (*p* = 0.033) but not at other time-points or in merged data (Table 1). There were no significant correlations between CV-VC and skin temperature (*p* > 0.05) (Table 1).

**TABLE 1:** Correlations of relative changes in conduction velocity during evoked contraction (CV-EC) and voluntary contraction (CV-VC) with relative changes in skin temperature.

**Relative changes in CV-EC vs. skin temperature**			**Relative changes in CV-VC vs. skin temperature**		
	***r***	***p***		***r***	***p***
POST	−0.62	0.054	POST	−0.07	0.867
POST30	−0.32	0.365	POST30	−0.67	0.071
POST60	−0.24	0.511	POST60	0.04	0.939
POST120	−0.67	0.033*	POST120	−0.39	0.383
Post24H	0.05	0.881	Post24H	−0.67	0.071
Merged	0.12	0.401	Merged	0.62	0.083

*p < 0.05.

**FIGURE 6:**
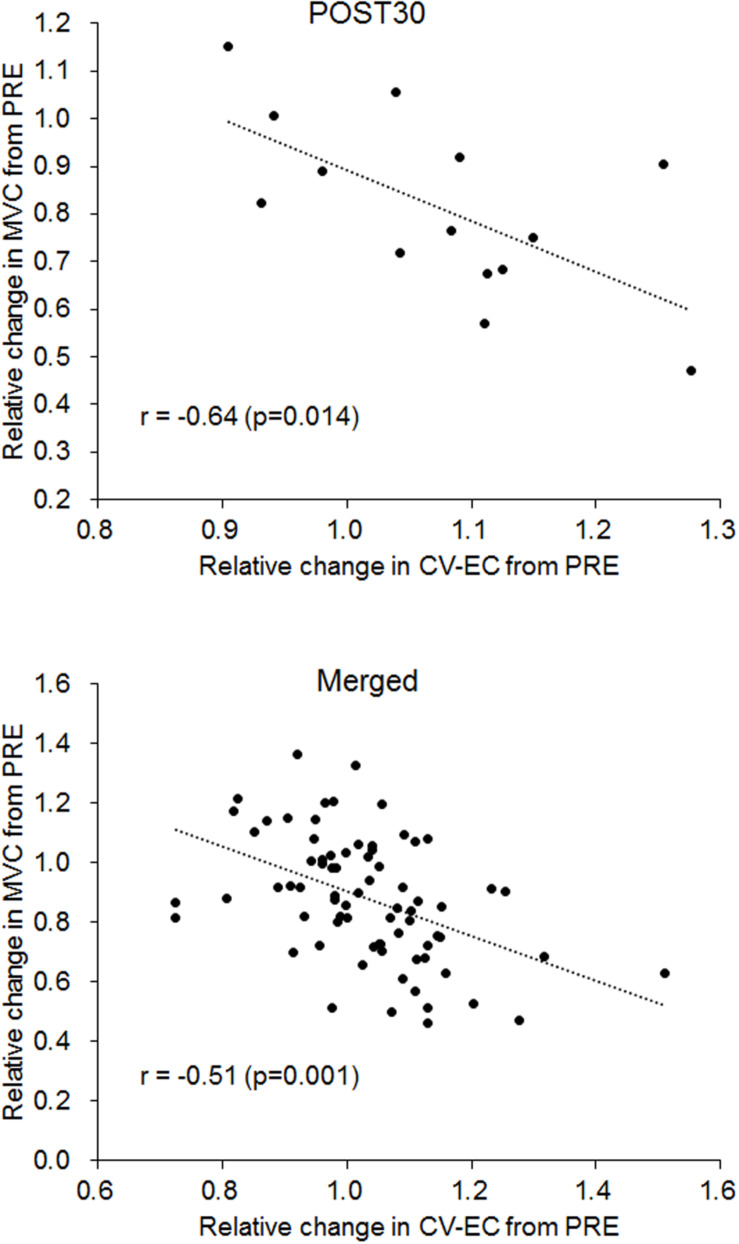
Correlation between relative changes in conduction velocity during evoked contraction (CV-EC) and maximal voluntary contraction (MVC) for 30 min after the fatiguing exercise (POST30) (upper panel) and for the merged data from all measurement periods (lower panel).

Significant correlations between CV-EC and MVC (relative values) were observed for POST30 and merged data (*p* < 0.05) (Figure 6 and Table 2). No significant correlations were noted between CV-VC and MVC (relative values) (*p* > 0.05) (Table 2).

**TABLE 2:** Correlations of relative changes in conduction velocity during evoked contraction (CV-EC) and voluntary contraction (CV-VC) with relative changes in maximal voluntary contraction (MVC).

**Relative changes in CV-EC vs. MVC**			**Relative changes in CV-VC vs. MVC**		
	***r***	***p***		***r***	***p***
POST	−0.50	0.069	POST	0.140	0.665
POST30	−0.64	0.014*	POST30	0.133	0.681
POST60	−0.33	0.246	POST60	0.427	0.190
POST120	0.01	0.970	POST120	0.000	1.000
Post24H	−0.05	0.864	Post24H	0.566	0.055
Merged	−0.51	0.001*	Merged	0.173	0.193

*p < 0.05.

## Discussion

The present study investigated time-course changes in CV of knee extensor muscle during EC and VC after fatiguing pedaling exercise. The main findings of this study were as follows: (1) CV during EC significantly increased immediately after the fatiguing exercise (Figure 4), (2) CV during VC significantly decreased 30, 60, and 120 min after the fatiguing exercise (Figure 4), (3) there were no significant positive correlations between CV and skin temperature, which is used as an indicator of the muscle temperature (Table 1), and (4) a significant negative correlation between CV during EC and MVC was observed (Figure 6). These results did not support our hypotheses that CV during EC remains unchanged and CV during VC increases after exercise and individual differences in CV changes could be explained by an indicator of muscle temperature when this indicator is altered by exercise. Also, CV during EC may be more useful when considering exercise-induced neuromuscular fatigue than CV during VC.

It has been reported that CV is rapidly decreased during maximal contractions and a sustained MVC induces neuromuscular fatigue (Bigland-Ritchie et al., 1981). Thus, CV during MVC would be affected by its contraction-induced decrease in CV and decrease in CV elicited by neuromuscular fatigue by itself. The results of CV during VC in our study could reflect not only differences in methodology to calculate CV, but also neuromuscular fatigue induced by the task itself.

In the present study, participants performed constant pedaling on a cycle ergometer at 70% of VO2 max until exhaustion as fatiguing exercise. MVC, which is used as a major indicator of neuromuscular fatigue, was significantly decreased immediately (*p* = 0.005) and 60 min after the exercise (Figure 3). Previous studies using a similar method of CV detection showed an approximately 10–30% reduction of MVC immediately after repeated concentric elbow flexion, repeated eccentric elbow flexion, cross-country skiing, and a half-marathon, respectively (Piitulainen et al., 2011; Boccia et al., 2017a, b). The present study showed an approximately 28% reduction of MVC immediately after the exercise. Also, the blood lactate concentration significantly increased immediately (*p* = 0.005) and 30 min (*p* = 0.01) after the exercise. These results indicate that the fatiguing exercise used in the present study induced neuromuscular fatigue in the participants. In the previous studies that used similar protocol, reductions in MVC after cycling exercise until exhaustion at 75% and 70% of VO2max were 34% and 29% (Sahlin and Seger, 1995; Presland et al., 2005). Since reduction of MVC in the present study was 28%, we think the degree of fatigue in this study may be comparable to that of previous studies.

Conduction velocity during EC increased immediately after the exercise (*p* = 0.005) (Figure 4). Since it is well-known that CV is influenced by muscle temperature (Petrofsky and Lind, 1980; Petrofsky and Laymon, 2005), an increase in CV could generally be explained by a corresponding increase in muscle temperature. We detected an increase in skin temperature, which was measured as an index of a change in muscle temperature, immediately and 30 min after the exercise (Figure 5), but no positive correlations between CV during EC and skin temperature were detected in this study (Table 1). From this result, we couldn’t explain an increase in CV-EC by an increase in muscle temperature. However, this result must be interpreted with caution, because detection of skin temperature was performed in small sample size (*n* = 10) and skin temperature is not direct measurements of muscle temperature. Skin temperature measured by thermography has been classically applied after exercises (Clark et al., 1977) and has been used to estimate use of skeletal muscles (Chudecka et al., 2015). In the previous study, during skin cooling, change in skin temperature are relatively similar with change in intramuscular temperature at 1 cm below a skin, but not those at 2 and 3 cm below a skin (Enwemeka et al., 2002). This suggests that changes in muscle temperature within 1.0 cm below a skin could be reflected in skin temperature. In our previous study (Watanabe et al., 2012), subcutaneous tissue thickness measured by ultrasonography at the lateral side of thigh was 0.54 cm (SD: 0.18) for young male. Although we didn’t measure subcutaneous tissue thickness from the participants of the present study, it can be estimated that the distance from skin surface to superficial borders of the vastus lateralis muscle is lesser than 1 cm from the result of the previous study (Watanabe et al., 2012). Therefore, changes in skin temperature at the measured sites in the present study could reflect changes in a part of muscle temperature. On the other hand, measuring a skin temperature is strongly influenced by some factors in addition to body temperature including muscle temperature. Crawford et al. (2006) stated that skin temperature reading is lower by perspiration or if skin is covered with something such as oils or hair is obstructing the recording (Crawford et al., 2006). They also described that the environmental conditions need to be carefully controlled to minimize errors such as draught-free neutral temperature environment (18–24°C). Although it was difficult to control perspiration of the participants, the measurement points around the electrodes on thigh was shaved and measurement was performed in laboratory with draught-free condition in the present study. As mentioned above, room temperature in the present study was 24.1 ± 1.3°C and this would be slightly higher than neutral temperature environment that is defined in Crawford et al. (2006). Therefore, while we minimized the factors influencing the skin temperature measurement, we should note that our methodology to estimate muscle temperature included the above-mentioned methodological limitations.

Conduction velocity during VC significantly decreased at 30, 60, and 120 min after the exercise, while no significant change in CV during VC was detected immediately after the exercise (Figure 4). This delayed onset of a decrease in CV or other related EMG parameters was also noted in previous studies (Piitulainen et al., 2011; Schmitz et al., 2012). For example, Piitulainen et al. (2011) detected a significant decrease in CV during MVC 2 h after repeated eccentric exercise, but not immediately after the exercise (Piitulainen et al., 2011). Also, they showed that the myoglobin concentration, which is an indicator of sarcolemmal permeability, exhibits the highest values at 2 h after fatiguing exercise among the time-points of immediately, 2 h after, and 24 h after exercise. This sarcolemmal permeability is considered to be one of the factors inducing changes in the muscle membrane condition (Mortimer et al., 1970; Moxham et al., 1982; Milner-Brown and Miller, 1986). Moreover, although the time-course was short and direct comparison with our study would be difficult, Schmitz et al. (2012) showed that a decrease in the pH within quadriceps muscles was observed with a time-lag, and CV decreased in parallel with changes in pH during exhaustive bicycling (Schmitz et al., 2012). Therefore, we speculated that the time-course of CV-VC in the present study may be in line with previous studies and can be explained by the physiological process occurring after exhaustive exercise. However, since types, intensity, or duration of the given exercise in the present study were not consistent with the previous studies, it would be difficult to conclude that changes in CV is occurred with same physiological phenomena reported in the previous studies.

From the results of this study, we suggest that CV during EC and VC exhibits different time-course changes after fatiguing pedaling exercise. While it was difficult to fully understand the mechanisms leading to the increase in CV-EC immediately after the exercise, CV-EC was associated with MVC, which is widely used to assess neuromuscular fatigue. We found significant negative correlations between the relative changes in CV-EC and MVC in the data at 30 min after the exercise and the merged data among the time-periods (*r* = −0.640, *p* = 0.014 for 30 min after the exercise; *r* = −0.511, *p* < 0.001 for the merged data) (Figure 6 and Table 2). On the other hand, no significant correlations between CV-VC and MVC were detected in any of the time-periods or merged data in the present study (*p* > 0.05) (Table 2). Since CV-EC was measured during EC, not during VC, it was independent of the performed force and/or physiological conditions during VC. This result suggests that CV measured during EC can be used to estimate MVC or fatigue conditions in the neuromuscular system without VC. However, we should note that CV-EC in this study mainly reflected physiological conditions of peripheral muscle components since electrical stimulation was applied to the estimated innervation zone. Also, considering that CV during VC decreases after fatiguing exercise, an increase in CV during EC after exercise may counteract the results of CV during VC.

## Conclusion

In conclusion, we investigated the time-course changes in CV of the knee extensor muscle during EC and VC after fatiguing pedaling exercise. CV during EC and VC significantly increased immediately after the fatiguing exercise and significantly decreased 30, 60, and 120 min after the fatiguing exercise, respectively. Also, a significant negative correlation between CV during EC and MVC was observed. We suggest that CV during EC and VC exhibited different time-course changes, and that CV during EC may be appropriate to estimate the degree of neuromuscular fatigue after fatiguing pedaling exercise.

## Data Availability Statement

The raw data supporting the conclusions of this article will be made available by the authors, without undue reservation, to any qualified researcher.

## Ethics Statement

The studies involving human participants were reviewed and approved by the Committee for Human Experimentation of Ritsumeikan University. The patients/participants provided their written informed consent to participate in this study.

## Author Contributions

KW, NHR, MY, NHG, MU, and MI designed the study. KW, TS, SK, NHZ, NHR, MY, NHG, KI, KT, MU, MK, and MI measured and collected the data. KW, TS, and MI analyzed the data and wrote the main parts of the manuscript. KW, TS, SK, NHZ, NHR, MY, NHG, KI, KT, MU, MK, and MI reviewed the manuscript.

## Conflict of Interest

The authors declare that the research was conducted in the absence of any commercial or financial relationships that could be construed as a potential conflict of interest.
